# The Role of Parental and Institutional Approaches in the Persistence of Pediculosis Capitis in Early Childhood Education Settings: A General Survey

**DOI:** 10.3390/insects16030308

**Published:** 2025-03-16

**Authors:** Marzena Kotus, Aleksandra Sędzikowska, Joanna Kulisz, Zbigniew Zając, Agnieszka Borzęcka-Sapko, Aneta Woźniak, Andrzej Tytuła, Katarzyna Bartosik

**Affiliations:** 1 Department of Anaesthesiological and Intensive Care Nursing, Faculty of Health Sciences, Medical University of Lublin, ul. Chodźki 7 St., 20-093 Lublin, Poland; marzena.kotus@umlub.pl; 2 Chair and Department of General Biology and Parasitology, Faculty of Medicine, Medical University of Warsaw, Chalubinskiego 5 St., 02-004 Warsaw, Poland; aleksandra.sedzikowska@wum.edu.pl; 3 Department of Biology and Parasitology, Faculty of Health Sciences, Medical University of Lublin, Radziwiłłowska 11 St., 20-080 Lublin, Poland; joanna.kulisz@umlub.pl (J.K.); zbigniew.zajac@umlub.pl (Z.Z.); aneta.wozniak@umlub.pl (A.W.); 4 NZOZ Med.-Laser, Młyńska 14 St., 20-406 Lublin, Poland; agnieszka.borzecka@op.pl; 5 Faculty of Human Sciences, WSEI University, 20-209 Lublin, Poland; a.tytula@nipip.pl

**Keywords:** *Pediculus humanus capitis*, head louse infestation, pediculosis capitis, arthropod bites, occupational infestation, familial pediculosis, stigma, kindergartens, preschools, Poland

## Abstract

*Pediculus humanus capitis* infestation is one of the most underestimated parasitoses still constituting a current epidemiological and social problem. Haematophagous insects, the etiological factor of pediculosis capitis, spread most easily in preschool children, which is determined by social and behavioural aspects. The study aimed to search for factors contributing to pediculosis capitis persistence in kindergartens located in south-eastern Poland. For this purpose, anonymous surveys among the management staff of preschool institutions were conducted. Head lice infestations were reported in 87.0% of the 561 surveyed facilities. A positive correlation was confirmed between the prevalence of head lice and a higher number of children in these facilities. Additionally, in 69.3% of the facilities where cases of pediculosis capitis were reported, it was noted that the infestation was associated with the child’s attendance at preschool. Familial and occupational transmission was observed in 78.5% and 25.2% of the facilities, respectively. In contrast, in 93.2% of preschools where no cases of infestation were reported, all parents consented to periodic head inspections. Parents’ attitudes and their perception of head lice as a health issue, rather than an embarrassing problem, appear to be key factors in effectively limiting the spread of head lice in the studied kindergartens.

## Simple Summary

Head louse infestation is still a current epidemiological and social issue worldwide. Cases of pediculosis capitis in most countries are not subject to registration, which prevents a reliable estimation of the prevalence of this ectoparasitosis. Parasitic insects spread most easily in close-contact environments, which are common in preschools, and contribute to disease persistence. This study aimed to search for behavioural determinants of pediculosis capitis occurrence in kindergartens, which could indicate a low-cost strategy to limit the persistence and spread of this infectious disease. Although pediculosis capitis is a common health issue among preschool children in Poland, collaboration between parents and educational institutions still needs improvement. Stigma related to head lice infestation seems to be a key factor facilitating disease persistence in preschool educational settings.

## 1. Introduction

Pediculosis capitis is a common neglected infectious disease affecting mainly school and preschool children in various regions of the world [1,2,3,4]. The etiological factor of the disease is the haematophagous insect *Pediculus humanus capitis* De Geer 1767 (Phthiraptera: Pediculidae), which is a permanent ectoparasite of humans.

Infestation with head lice affects residents of both rural and urban areas [2,5,6]. Although the parasitosis is observed in all seasons, the increase in its prevalence is seasonal and is often observed after days off from school and preschool educational facilities [7,8,9]. The prevalence of pediculosis capitis may be influenced by socio-demographic and economic factors, e.g., the family size, housing conditions, educational level, or family income [6,7,10,11,12]. In most countries, pediculosis capitis cases are not registered, which makes it impossible to reliably assess the incidence of this infectious disease. However, pediculosis is still a serious social problem generating high treatment costs, which annually reach approximately USD 500 million in the USA or USD 5,790,143 in Iran [13,14].

Adult head lice are 2–4.7 mm long wingless insects living close to the surface of the scalp, where they are provided with food, appropriate temperature and humidity [15,16]. The female lays about 3–9 eggs per day; attached to the hair by cement, which hardens into a sheath, protecting the embryo developing in the egg from mechanical damage and dehydration [17,18]. The female, which lives for approximately a month, produces between 55 and 210 eggs during the lifespan. The oviposition is influenced by, e.g., the age of the female and the frequency of feeding [19,20,21]. Of all active developmental stages of *P. humanus capitis*, adults exhibit the highest tendency to disperse and find a new host [22]. Head lice can be transmitted both directly [23] and indirectly (with a lesser epidemiological significance of the latter route), e.g., through pillowcases or sharing combs and other hair accessories, such as barrettes or elastic bands, where lice can survive for several hours [20,24,25]. Due to their close dependence on the host, specimens of *P. humanus capitis* can live off the host for up to 36 h, and their eggs can survive for up to 10 days, depending on abiotic conditions, i.e., temperature and relative humidity [25]. As reported by Burgess [26], a louse can migrate from one head to another within approximately 30 s. A study on spatial and kinetic factors that may affect the transmission of *P. humanus capitis* between hairs conducted by Canyon et al. [27] demonstrated that head lice migrate from one hair to another most efficiently when the hairs are in parallel arrangement during prolonged close head-to-head contact. The hair-to-hair transmission is also favoured by the position of the lice, i.e., above the host’s scalp with their abdomen tip facing the host’s head [27].

Pediculosis capitis spreads most easily among preschool and early-school children. Literature data indicate that the highest pediculosis capitis incidence is noted in children between 2 and 12 years of age [3]. The high prevalence of infestation in this age group is associated primarily with social behaviour, i.e., prolonged close contact during playing, which facilitates head louse transmission during school attendance. The infestation is more frequent among girls than among boys, which may be related both to the fact that girls have long hair more often than boys [28,29,30,31] and to the phase of psycho-social development, in which children prefer interactions with same-sex peers [32]. Moreover, in the case of long hair, low-intensity infestation may be overlooked, thus allowing the parasite to increase in number, and the eradication of these insects poses a greater challenge [9,11,33]. As reported by Gallasi et al., volatile compounds constituting the head odour serve as insect attractants more than the odours of other body parts [34]. It has also been demonstrated that the combination of three major volatile components present in the human scalp (nonanal, sulcatone, geranylacetone) serves as an attractant for head lice [35].

Similarly to other solenophages (vessel feeders), head lice counteract the haemostatic mechanisms of the host via anticoagulant and anti-platelet agents [36]. Lice feed several times a day for approximately 3–15 min [37,38]. In the case of prolonged exposure to insect bites, there is a risk of developing sensitisation to the components of louse saliva and/or faeces [39,40,41].

**Figure 1:**
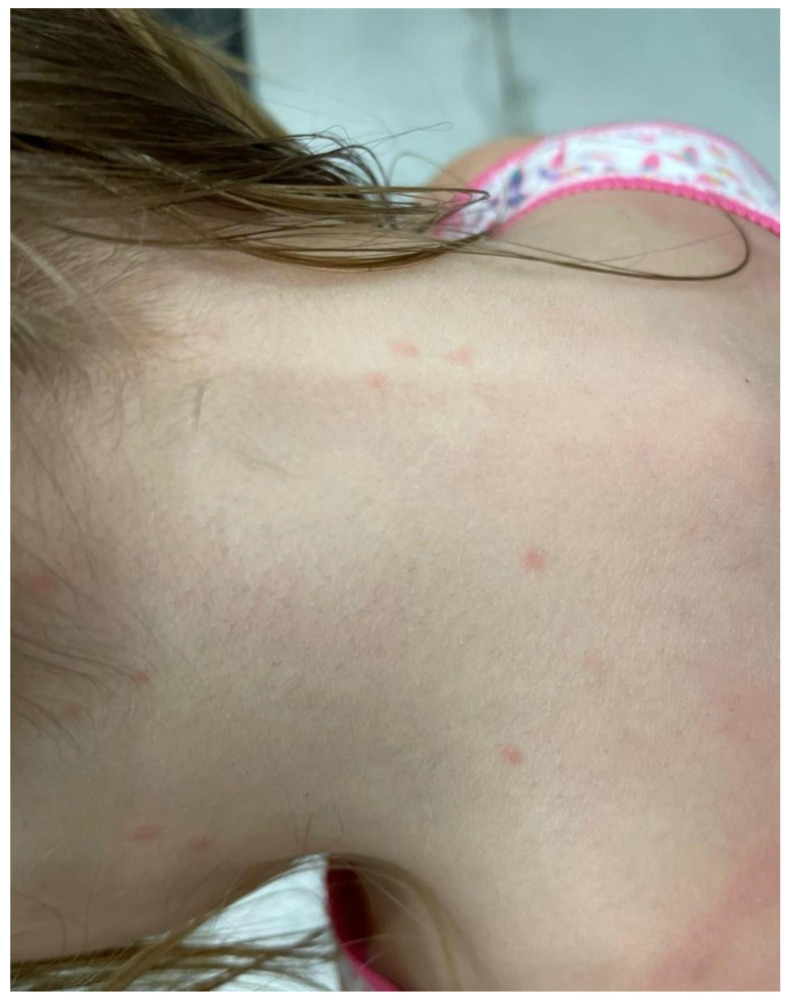
Pediculosis capitis in a 7-year-old girl acquired from her 3-year-old brother attending kindergarten; erythematous skin lesions accompanied by pruritus and red spots at the site of head louse bites in the occipital region and on the jaw, neck, and nape (Photograph: Agnieszka Borzęcka-Sapko).

**Figure 2:**
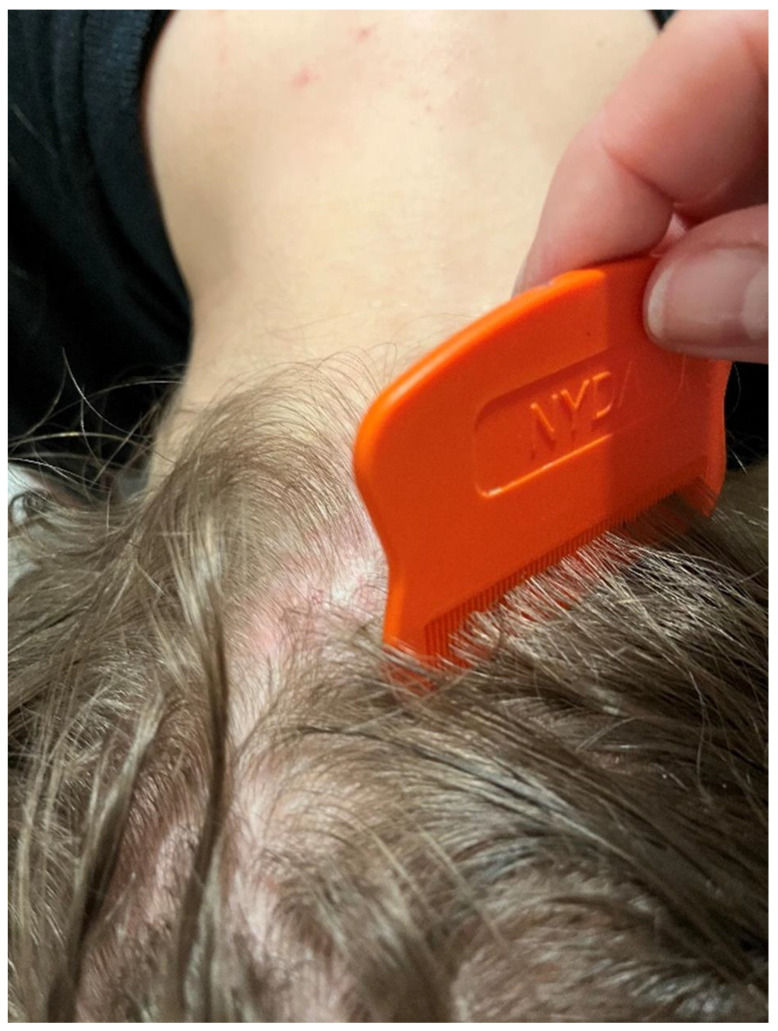
Irregularly shaped erythematous skin lesions, accompanied by pruritus, clearly demarcated from healthy skin with accompanying severe itching in the course of *Pediculus humanus capitis* infestation in a previously exposed child (Photograph: Katarzyna Bartosik).

Severe itching associated with sensitisation appears after 4-6 weeks or more rapidly in re-infestations [42,43] and may persist for several weeks or even months after effective treatment [44,45]. During the infestation, erythematous skin lesions accompanied by pruritus are noted on the scalp, neck, and nape (Figure 1 and Figure 2). The skin lesions caused by head lice are mostly located in the occipital and temporal regions, where oozing lesions develop, often with impetiginisation [46]. Repetitive scratching by a person infested by lice may lead to loss of skin integrity and, consequently, secondary bacterial infection [47]. Severe suppurative inflammation of the scalp may cause the development of irregular patches of scarring alopecia [48]. Some patients may experience fever, malaise, irritability, and enlargement of cervical and occipital lymph nodes [49]. A severe head louse infestation in school-age children may lead to iron deficiency anaemia [38]. Allergic reactions, e.g., rhinoconjunctivitis or diffuse autoeczematisation involving extremities and the torso accompanied by severe crusting and scaling of the scalp, have also been reported in the course of pediculosis capitis [39,40,41,50,51].

According to the data from the Central Statistical Office in Poland, on 30 September 2019, 90.4% (1.4 million) of children aged 3–6 years participated in various forms of preschool education. In the study area, children covered by the first stage of education constituted 86.5–89.3% of all children aged 3–6 years, i.e., 70,165 children in Lubelskie Voivodeship and 74,296 in Podkarpackie Voivodeship [52].

Likewise, Poland, at the time of the study, had no legal basis for the mandatory employment of nurses in care and educational facilities, and preventive measures to combat head lice were part of grassroots action undertaken by directors of facilities, teachers, educators, parents, and nurses working in the teaching and educational environment.

The Act of 14 December 2016 on Education Law (Journal of Laws 2018, items 996 and 1000) imposes an obligation on educational facilities to provide organisational settings for safe and hygienic conditions of the stay of children in these facilities, regardless of whether or not the facilities employ a nurse. Therefore, the burden of combating this parasitosis and anti-epidemic activities have been shifted from the State Sanitary Inspectorate to educational and care facilities for children.

The aim of the study was to assess the prevalence of pediculosis in preschool children, determine factors contributing to the presence and spread of the parasitosis in preschools, and identify actions implemented to prevent and reduce the prevalence of head lice in children and preschool employees in Lubelskie and Podkarpackie Voivodeships.

## 2. Materials and Methods

**Figure 3:**
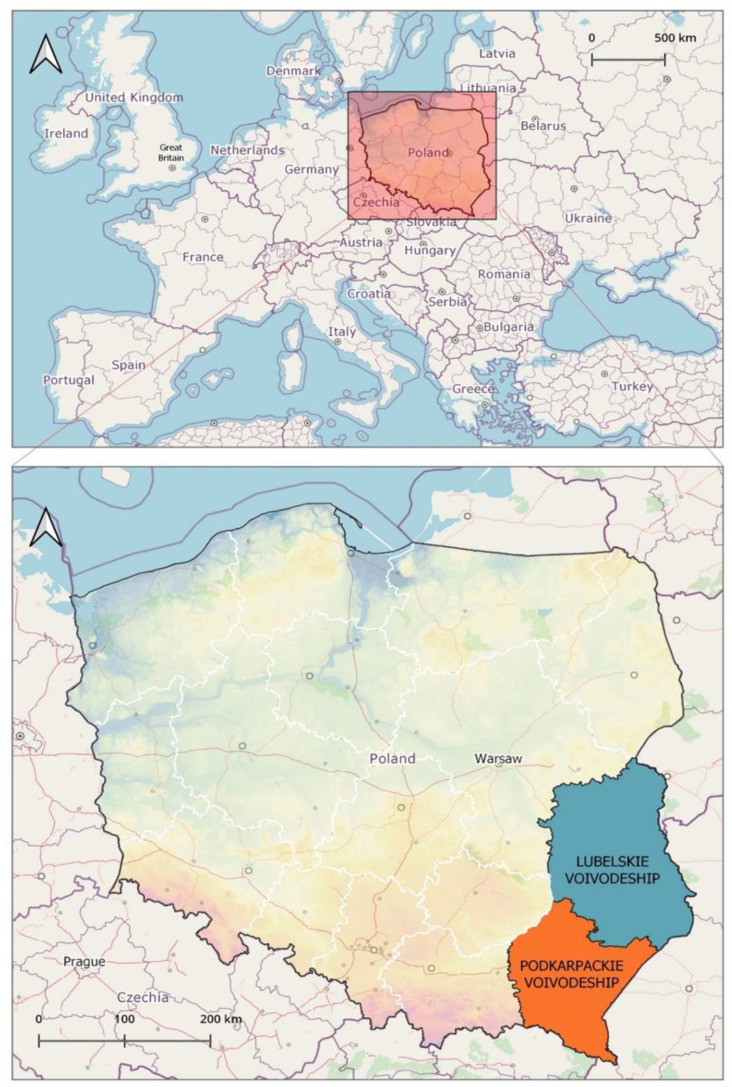
Area of the study in south-eastern Poland (prepared by Marcin Wasilewski, marcinwasilewski.eu on the basis of the OpenStreetMap; © authors OpenStreetMap).

The data for the analysis of the occurrence of pediculosis in preschool children were collected during a retrospective study conducted in Lubelskie and Podkarpackie Voivodeships, south-eastern Poland, in 2019 (Figure 3). The research method was based on a diagnostic survey, with an author’s questionnaire used as a research tool.

The study was based on a survey addressed to the managers of preschool facilities and consisted of 24 questions about such information as the size of the town/city in which the preschool is located (population size), the presence of pediculosis in the preschool in the last 5 years (2014–2018), the age and gender of infested children, re-infestations in children, familial and occupational transmission of head lice, methods of communication between the facility and parents, subjective assessment of the perception of head louse infestation as embarrassing, and subjective assessment of the level of difficulty in limiting the prevalence of pediculosis in the facility. The study also included data on educational campaigns focusing on the presence and frequency of pediculosis, addressees of the campaigns, and the person responsible for educational activities in the preschool. It was also conducted to determine the dynamics of pediculosis prevalence in the studied facilities and the support from other institutions, such as the education authority, the sanitary–epidemiological station, and the Ministry, in the fight against pediculosis in children. The surveys were delivered to the directors of the facilities in person, electronically (e-mail, Google form), and by traditional mail.

The participation in the study was voluntary. The respondents were informed about the aim of the study. The investigators also provided substantive support upon request from the surveyed directors of the facilities. The criterion for inclusion in the study was the provision of preschool education (for children aged 3–6) in the study area since 2014, regardless of whether the kindergarten was a public or private facility.

The data obtained from the surveys were subjected to statistical analysis. The Pearson chi-square test was used to examine the relationship between the variables, and the test for significance of the differences between two indicators was employed. When the variables did not have a normal distribution or were discrete, the Mann–Whitney U test was used to check the significance of differences in the variables between two groups. A value of *p* < 0.05 was considered statistically significant. Statistical calculations were performed using the STATISTICA 10 PL statistical package.

## 3. Results

In total, 561 questionnaires filled out by the directors of kindergarten facilities were analysed in the study. All of the 276 questionnaires delivered in person to the preschool representatives were completed, i.e., the questionnaire return rate (the effectiveness of data collection using this method) was 100%.

Of the 596 surveys sent electronically, 237 were completed by the directors of the care and educational facilities, i.e., the return rate was 39.8%. Of the 75 surveys sent to preschools by traditional mail, 48 completed surveys were returned, i.e., the return rate was 64%.

The results showed that, during the last five years preceding the study (2014–2018), pediculosis was reported in 87% (488/561) of the surveyed preschools. The mean infestation prevalence in these facilities was 1.18 ± 1.23%. Moreover, in 69.3% (338/488) of facilities where pediculosis was diagnosed, the presence of the infestation was associated with the child’s attendance to the preschools. The Mann–Whitney U test confirmed the positive correlation between the prevalence of pediculosis capitis and the number of children in the care and educational facilities (Z = 7.41; *p* < 0.0001) (Table 1). The study also showed a relationship between the prevalence of pediculosis and the size of the town/city where the kindergarten was located (χ^2^ = 49.44; *p* < 0.0001) (Table 2).

Among children with diagnosed pediculosis capitis, 60.7% were 5–6 years old, while 39.3% were 3–4 years old. Additionally, a statistically significant difference (*p* < 0.0001) was found in the frequency of this parasitosis between boys and girls, i.e., infested girls were recorded in 94.7% (462/488) of facilities where pediculosis was detected, while infestation cases in boys were registered in 66.4% (324/488) of the facilities.

Reinfestations diagnosed in the same child, were recorded in 60.7% (296/488) of facilities where pediculosis was detected. In turn, familial infestations, including transmission between siblings and between children and parents, were recorded in 78.5% (383/488) and 33.8% (165/488) of the preschools, respectively. Occupational transmission of the parasitosis, i.e., between the children and preschool staff, was reported in 25.2% (123/488) of facilities where pediculosis capitis cases were recorded.

The present analyses revealed a significant correlation between the parental consent to have their children’s heads inspected and the presence of pediculosis in the preschool (χ^2^ = 15.80; *p* < 0.0001). In 93.2% of preschools where no pediculosis was detected, all parents gave consent to have their children’s heads inspected (Table 3).

The statistical analysis of the data obtained using the Pearson chi-square test showed a significant relationship between the parental consent to inspect children’s heads and the size of the town/city (χ^2^ = 32.72; *p* = < 0.0001). In towns with less than 10 thousand residents and cities with a population size over 500 thousand, the percentage of preschools where not all parents gave their consent for inspection of their child’s head was higher, i.e., 31.3% and 55.3%, respectively. In towns/cities with 10–50 thousand, 50–100 thousand, and 100–500 thousand inhabitants, there were relatively fewer preschool facilities in which not all parents agreed to the hygiene checks, i.e., 12%, 18.3%, and 25%.

This study also showed that the preschools employed various methods of informing parents about the occurrence of pediculosis capitis. The most frequently chosen methods included individual phone conversations with parents (73.2%), information in direct contact between the teacher and the child’s parent/guardian (62.1%), and posting the information about the occurrence of pediculosis on the preschool notice board (60.0%). The statistical analysis revealed significant differences between the methods used by the facility staff to inform parents, depending on the size of the town/city where the facility was located. The method of posting the information on a notice board (*p* = 0.0030) was most frequently chosen in preschools located in towns/cities with 10–50 thousand inhabitants and over 500 thousand inhabitants, i.e., 71.9% and 68.1%, respectively. In turn, the method of direct contact between a teacher and a parent/guardian (*p* = 0.0028) was chosen mainly in preschool facilities located in towns/cities with 10–50 thousand inhabitants and over 500 thousand inhabitants (89.5% and 76.6%, respectively). Moreover, in towns inhabited by up to 10 thousand residents, 34.9% of the facilities most frequently informed parents by telephone (*p* = 0.0012).

The collected data showed that 69.2% (388/561) of the analysed preschools provided educational campaigns as part of the prophylaxis of pediculosis capitis, which were mainly intended for parents (86.9% of the facilities) but to a lesser extent for children (51.3%) and preschool employees (51.0%).

The educational and informational campaigns provided by the facilities exhibited a significant correlation with the size of the town/city where the preschool was located (*p* = 0.0001). A higher percentage of preschools where the educational campaigns on pediculosis were conducted were recorded in cities with 50–100 thousand inhabitants (12.9%) and over 500 thousand inhabitants (10.8%). In turn, in cities with 100–500 thousand inhabitants, 63.0% of the examined facilities did not organise such campaigns. The analysis also revealed significant differences in the frequency of preventive activities undertaken in the facilities located in towns/cities with different population sizes. In each of the five size categories, the largest percentage of facilities declared such activities on an as-needed basis, i.e., when pediculosis was diagnosed in the children (Table 4).

The present study showed that pediculosis was perceived as an embarrassing issue in 56.1% (274/488) of the examined facilities. Moreover, the analysis of the results showed a correlation between the method of informing parents about the occurrence of pediculosis and the perception of the infestation as an embarrassing issue by the facility staff. The method of individual contact between the teacher and the parent was employed more often (79.2%) in preschools that considered pediculosis an awkward issue (*p* = 0.0007), whereas e-mailing multiple recipients, i.e., a group of parents (*p* = 0.0461), was more often (15.4%) used as a method of information in facilities where pediculosis was not regarded as a natural/usual health issue. A significant correlation was also found between the rate of parental reporting to preschool staff about pediculosis in their children and the perception of the parasitosis as a shaming issue (*p* = 0.0037). Sporadic parental reports on the presence of pediculosis in their children were declared by a higher percentage of facilities where pediculosis was perceived as an embarrassing problem (50.9%) (Table 5).

Consent from all parents for the child’s head inspection was obtained by 67.8% of facilities where the head louse infestation was perceived as a shaming problem and by 82.6% of preschools where the parasitosis was treated as a non-embarrassing health issue. The Pearson chi-square analysis of the results revealed a significant correlation between the parental consent to examine their children’s scalp and the perception of pediculosis as an embarrassing topic by the facility staff (χ^2^ = 15.63; *p* < 0.0001).

A significant correlation was found between parental reporting to preschool staff on the louse infestation in their children and the size of the town/city where the facility was located (*p* < 0.0001). In smaller towns, below 10 thousand inhabitants and those with between 10 and 50 thousand inhabitants, the preschools where parents did not report pediculosis accounted for a high percentage, i.e., 20.6% and 26.5%, respectively (Table 6).

The size of the town/city where the preschools were located showed a significant correlation with the dynamics of pediculosis occurrence in the period of 5 years preceding the study (*p* = 0.0034). The percentage of preschools where the number of pediculosis cases decreased was higher in towns/cities with 10–50 thousand (58.8%), 50–100 thousand (54.3%), and over 500 thousand (62.5%) of residents. In turn, 68.5% of preschools located in cities with 100–500 thousand residents reported an upward trend in the occurrence of pediculosis in this period.

An increasing number of pediculosis cases was reported more frequently in facilities with higher values of the mean (142.8) and median (129.0) number of children, and the Mann–Whitney U test confirmed that this relationship was statistically significant (Z = 2.27; *p* = 0.0234). Additionally, a significant relationship was demonstrated between the perception of pediculosis as a shaming issue and the dynamics of pediculosis occurrence in the analysed preschools (χ^2^ = 29.27; *p* = < 0.0001). In 69.9% of facilities where pediculosis was perceived as an embarrassing issue, the number of pediculosis cases increased during the study period.

The analysis of the results (multiple responses) showed significant differences in terms of the persons involved in the pediculosis prophylactic activities between the facilities located in towns/cities with different numbers of inhabitants. The percentages of preschools where such activities were conducted by a teacher or employees of sanitary–epidemiological stations did not differ significantly relative to the size of the town/city. In contrast, significant differences between preschools located in towns/cities with different numbers of inhabitants were found in the case of the response “nurse” (*p* = 0.0084) chosen as the person who conducted prophylactic activities in towns/cities with less than 10 thousand (54.5%) and with 50–100 thousand inhabitants (58.0%).

The Pearson chi-square test also revealed a significant relationship between the location of the preschool and the institutional support in pediculosis prophylactic and diagnostic activities offered to the facilities (χ^2^ = 23.77; *p* < 0.0001). As many as 60.2% of the facilities in towns with less than 10 thousand residents and 63.2% of preschools in cities with 100–500 thousand inhabitants did not receive any of the aforementioned support (Table 7).

The control of pediculosis in the preschools was perceived as difficult by the staff of 121 facilities (24.7%) out of the 488 preschools where the parasitosis was detected. The statistical analysis showed a significant positive correlation between the lack of institutional support and the declaration of difficulties in the control of the pediculosis prevalence in the analysed preschools (χ^2^ = 25.00; *p* < 0.0001). Additionally, in preschools (63.3%) where not all parents gave consent for the inspection of their child’s head, the pediculosis control was perceived as difficult (χ^2^ = 4.27; *p* < 0.0389). This study showed that the control of this health issue was declared to be difficult in 69.1% of preschools where pediculosis was perceived as an embarrassing problem (*p* < 0.0001).

The study also revealed a significant relationship between preschools where the pediculosis control was perceived as difficult and the lack of information from parents about the infestation in their children (*p* = 0.0002). Moreover, the analysis of the results showed a significant relationship between the transmission of pediculosis capitis between siblings and reporting the problem to the facility by parents (*p* = 0.0163). In total, 81.0% of preschools where cases of familial transmission were recorded were informed about the problem by parents.

## 4. Discussion

Pediculosis capitis is still an important health issue for institutions responsible for health care, epidemiology, and health education [3,10,53,54,55,56,57]. In some regions of the world, the recorded prevalence of the parasitosis is very high, reaching even several tens of percent, e.g., 61.4% in Argentina [58] and 74.24% in Pakistan [59]. The importance of pediculosis capitis as an epidemiological and social problem in Poland is emphasised by its high prevalence (12.03%), as reported by Pazik et al. in their study based on direct inspection of 2060 preschool children in Warsaw, Krakow, and Poznań, conducted from September 2018 to 28 February 2020 [60]. In the present study, as many as 87% of the 561 preschool facilities surveyed reported cases of this disease in the last 5 years, and the declared mean pediculosis prevalence among the children in facilities where the problem was detected was 1.18 ± 1.23%. Although the above-mentioned data come from different regions of Poland, they indicate a large discrepancy between declarative and empirical data, which confirms how many cases of the disease remain unrecorded/overlooked by care and educational institutions. More than half of the directors (52.2%) of the surveyed facilities declared that the number of noted pediculosis cases was stable over the last 5 years. Alarmingly, however, an upward trend was observed in 27.8% of the preschools, especially in cities with 100–500 thousand inhabitants, (χ^2^ =15.73; *p* = 0.0034).

As a disease spreading via direct contact, pediculosis capitis may persist in local foci of high prevalence [58,59,61], and the elimination of head lice is usually difficult due to both the biology of the parasite and the epidemiology of the disease [62]. The difficulty may also be related to the low level of knowledge and awareness of infested subjects and their immediate surroundings [10]. Studies conducted in various parts of the world have confirmed the impact of the COVID-19-related social isolation on the reduction in pediculosis capitis prevalence [60,63,64,65,66]. Thus, it has been clearly confirmed that social contacts, including those in educational institutions, support the persistence of pediculosis capitis foci and spread significantly.

Preschool children (3–6 years old) are at increased risk of head louse infestation [1,60,67] due to their long-term close contact with the peer group as well as the insufficient knowledge about the prophylaxis of parasitic diseases and poor development of self-care and personal hygiene skills.

The size of preschool groups and, therefore, the number of potential contacts established by children, are usually higher in cities, which was evidenced in this study by the significant relationship between the size of the town/city and the prevalence of pediculosis capitis (*p* < 0.0001). In another study conducted in the same period (2014–2018) in Polish primary schools, the mean number of pupils attending the facility was significantly correlated with the occurrence of pediculosis capitis in educational settings. The number of pupils in pediculosis capitis-affected and non-affected schools was 387 ± 285 and 234 ± 183, respectively (*p* = 0.0169) [4].

Effective control of pediculosis capitis is closely related to prevention, e.g., inspection of the scalp to find the developmental forms of the parasite, including eggs [68,69]. In 74.2% of the facilities studied, all parents consented to the inspection of their children’s heads. The analysis revealed a significant relationship between the size of the town/city and the lack of consent from all parents to the inspection of their children’s heads. The percentage of preschools where not all parents consented to the inspection was higher in towns with up to 10 thousand inhabitants. The most common method of head lice detection is the inspection of children’s heads in schools and preschools by appropriately trained nursing staff. It is currently believed that the most effective methods of detection of these ectoparasites is to comb out lice and nits using a special louse comb with a tooth spacing of 0.2 to 0.30 millimetres [45,70]. However, this method is difficult to implement in preschool facilities on a national or even regional scale.

Family members and anyone in prolonged close physical contact with an infested person must be examined. Treatment should only be administered to those in whom viable eggs, living nymphal, or adult lice are found on the scalp [45,71]. Therapy of pediculosis capitis can be challenging, even with the numerous products available on the market. The effectiveness of the treatment depends, to a large extent, on applying pediculicides according to the manufacturer’s instructions, which should be aligned with the lice’s life cycle. It is essential for all individuals infested with lice to receive treatment simultaneously. The timing of each treatment should be adjusted to the mechanism of action of the product used. For single-use medicinal products, e.g., those containing physically acting dimethicone, the scalp should be re-examined on the same day as the treatment and again after 10 days. If the treatment includes pediculicides that require two applications, e.g., permethrin of neurotoxic activity, the patient should be re-examined on the 7th and 10th days following the start of treatment. During re-examination, any remaining eggs on the hair must be removed using a fine-toothed comb with teeth spaced between 0.09 and 0.19 mm apart on thoroughly wet hair. This procedure is 90.5% effective in detecting lice in every developmental form and is vital for eliminating any surviving parasites [45,70,72]. If a follow-up check on the 10th day reveals living lice, it is advisable to switch to a different active ingredient or one with a different mechanism of action. To ensure safety, only preparations approved by health authorities should be used; for instance, in Poland, products must be approved by the Office for Registration of Medicinal Products, Medical Devices and Biocidal Products. Additionally, for a pediculicide to be considered effective in reducing head lice, its efficacy should be at least 85%, and mild side effects should occur in no more than 5% of treated patients [45].

Prophylactic measures, e.g., education of all groups at risk, are extremely important, given the route of transmission of head lice and the age of the children in the analysed facilities [10,61,73,74]. The frequency of employing preventive measures that precede the appearance of pediculosis may have a positive effect on reducing its prevalence, as shown by the significant correlation (*p* = 0.0093) observed in this study. In the group of facilities with pediculosis capitis educational campaigns conducted once during a school year, the percentage of preschools where the parasitosis was not reported was higher (59.4%). The frequency of the preventive activities in the analysed facilities also differed depending on the size of the town/city, i.e., the preventive activities were most often undertaken once a year (*p* = 0.0084) or when pediculosis had already been detected (*p* = 0.0026) in cities with over 500 thousand inhabitants and once a semester in cities with 100–500 thousand inhabitants (*p* = 0.0017). The necessity to undertake pediculosis prophylaxis measures is extremely important, as indicated by many authors. Interestingly, the increasing awareness of pediculosis in subjects/families that had been affected by this health issue prompted the need for more frequent, more thorough, and regular inspection of children’s heads and more effective use of insecticides [10,73,75].

The information about pediculosis cases from parents to preschool staff is important given the transmission routes and epidemiology of this disease. In the present study, 42.2% of the facilities declared receiving parental reports about every case of pediculosis in their children, while 45.6% of the preschools were informed sporadically. Unfortunately, in 12.1% of the parasitosis-affected facilities, no such information was provided by parents at all. A disturbing finding is that, in smaller towns (up to 10 thousand inhabitants), the information about pediculosis was either not provided or provided only sporadically (31.9% of the preschools). This indicates that, in local communities characterised by a lower level of anonymity of the child and their family, the presence of head lice in children may be stigmatised and hence concealed by parents [10,71,76,77]. Moreover, the analysis of the survey data showed a relationship between the perception of pediculosis as an embarrassing issue and the refusal to consent to preventive check-ups, the lack of reporting the presence of head lice in children, and the increasing trend of pediculosis prevalence in the analysed educational facilities. The communication between parents and preschool staff is crucial for the control of louse infestations in young children, as highlighted by other researchers [73,75]. The perception of pediculosis as an embarrassing health problem may impede early detection and elimination of its foci in the initial phase of development, thereby promoting the spread of these parasites in the population. In the facilities where pediculosis was perceived as an embarrassing issue, a significant relationship was observed with the method of dissemination of the information about the presence of the infestation. Individual conversations between teachers and parents (*p* = 0.0007) were chosen more frequently in preschools where pediculosis was perceived as an embarrassing problem, while information was more often provided via collective e-mails in preschools that did not regard this disease as a shaming issue (*p* = 0.0461). The information methods that protect the privacy of the infested child and his/her family can significantly reduce the stigma of infested subjects and have a positive impact on the communication between parents and preschool staff in the event of pediculosis recurrence or other health or educational problems. In contrast, the stigmatisation of pediculosis has both social and economic consequences and may lead to the development of increased resistance of the parasites to insecticides due to their excessive and inappropriate use [75,76,78].

## Figures and Tables

**Table 1:** Descriptive statistics of the number of children in the analysed preschools versus the occurrence of pediculosis capitis in the facility.

Pediculosis in the Preschool	n	Number of Children in the Facility					Z	*p*
		M	SD	Me	Min.	Max.		
Presence	488	147.0	71.5	128.0	5	375	7.41	<0.0001
Absence	73	88.6	34.2	90.0	18	150		

n—number of facilities, M—mean, SD—standard deviation, Me—median, Min.—minimum, Max.—maximum, Z—Mann–Whitney U test, *p*—probability level.

**Table 2:** Presence of pediculosis in the examined preschools versus the size of town/city where the facility is located.

Location of the Preschool in a Town/City	n	%	Pediculosis in the Preschool				χ^2^	df	*p*
			Presence		Absence				
			n	%	n	%			
<10,000 *	83	14.8	63	12.9	20	27.4	49.44	4	<0.0001
10–50,000	83	14.8	57	11.7	26	35.6			
50–100,000	60	10.7	54	11.1	6	8.2			
100–500,000	288	51.3	267	54.7	21	28.8			
>500,000	47	8.4	47	9.6	0	0.0			
Total	561	100.0	488	100.0	73	100.0			

*—number of inhabitants, n—number of facilities, χ^2^—Pearson chi-square test, df—degrees of freedom, *p*—probability level.

**Table 3:** Relationship between the presence of pediculosis capitis in the preschool and parental consent for inspection of the children’s heads to diagnose pediculosis.

Do All Parents Give Consent for Inspection of Their Child’s Head?	n	%	Pediculosis in the Preschool				χ^2^	df	*p*
			Presence		Absence				
			n	%	n	%			
Yes	416	74.2	348	71.3	68	93.2	15.80	1	<0.0001
No	145	25.8	140	28.7	5	6.8			
Total	561	100.0	488	100.0	73	100.0			

n—number of facilities, χ^2^—Pearson chi-square test, df—degrees of freedom, *p*—probability level.

**Table 4:** Frequency of organising information campaigns versus the population size and the significance of differences between two structural indicators.

Frequency of Information Campaigns	Town/City										*p*
	<10,000 * n = 55		10–50,000 n = 62		50–100,000 n = 50		100–500,000 n = 179		>500,000 n = 42		
	n	%	n	%	n	%	n	%	n	%	
1 × school year	25	45.5	27	43.5	14	28.0	52	29.1	22	52.4	0.0084
1 × semester	5	9.1	7	11.3	11	22.0	53	29.6	6	14.3	0.0017
as needed*	42	76.4	38	61.3	37	74.0	108	60.3	37	88.1	0.0026

*—number of inhabitants, n—number of facilities, *p*—significance of differences between two structural indicators, * in the case of disease occurrence.

**Table 5:** Relationship between parental reports to preschool staff on pediculosis in their children and the perception of the infestation as a shaming issue by the staff.

Do Parents Inform Preschool Staff About Pediculosis in Their Children?	Do You Find Pediculosis a Shaming Issue?				χ^2^	df	*p*
	Yes		No				
	n	%	n	%			
Yes	116	36.3	121	50.2	11.23	2	0.0037
Yes, but only some parents/sporadically	163	50.9	93	38.6			
No	41	12.8	27	11.2			
Total	320	100.0	241	100.0			

n—number of facilities, χ^2^—Pearson chi-square test, df—degrees of freedom, *p*—probability level.

**Table 6:** Size of town/city versus parental reports to preschool staff on pediculosis in preschool children.

Town/City	Do Parents Inform Preschool Staff About Pediculosis in Their Children?						χ^2^	df	*p*
	Yes		Yes, but Only Some Parents/Sporadically		No				
	n	%	n	%	n	%			
<10,000 *	40	16.9	29	11.3	14	20.6	42.35	8	<0.0001
10–50,000	24	10.1	41	16.0	18	26.5			
50–100,000	14	5.9	43	16.8	3	4.4			
100–500,000	139	58.6	116	45.3	33	48.5			
>500,000	20	8.4	27	10.5	0	0.0			
Total	237	100.0	256	100.0	68	100.0			

*—number of inhabitants, n—number of facilities, χ^2^—Pearson chi-square test, df—degrees of freedom, *p*—probability level.

**Table 7:** Size of town/city versus institutional support in the control of pediculosis capitis.

Town/City	Can the Preschool Be Supported by Other Institutions in the Fight Against Pediculosis?				χ^2^	df	*p*
	Yes		No				
	n	%	n	%			
<10,000 *	33	13.2	50	16.1	23.77	4	<0.0001
10–50,000	45	18.0	38	12.2			
50–100,000	36	14.4	24	7.7			
100–500,000	106	42.4	182	58.5			
>500,000	30	12.0	17	5.5			
Total	250	100.0	311	100.0			

*—number of inhabitants, n—number of facilities, χ^2^—Pearson chi-square test, df—degrees of freedom, *p*—probability level.

## Data Availability

The original contributions presented in the study are included in the article; further inquiries can be directed to the corresponding author.
